# Cesarean section following idiopathic rupture of renal artery aneurysm leading to fetal dysfunction

**DOI:** 10.1186/s40981-019-0237-y

**Published:** 2019-03-06

**Authors:** Misa Matsuoka, Takashi Suto, Sayaka Hio, Shigeru Saito

**Affiliations:** 1 Department of Anesthesiology, Gunma University Hospital, 3-39-15, Showa-machi, Maebashi-shi, Gunma 371-8511 Japan; 2 Department of Anesthesiology, Gunma University Graduate School of Medicine, 3-39-22, showa-machi, Maebashi-shi, Gunma 371-8511 Japan; 3 Intensive Care Unit, Gunma University Hospital, 3-39-15 Showa-Machi, Maebashi-shi, Gunma 371-8511 Japan

**Keywords:** Rupture of renal artery aneurysm, Cesarean section, Fetal dysfunction, Transcatheter arterial embolization

## Abstract

**Background:**

Renal artery aneurysms (RAAs) in pregnancy are uncommon, with most found after rupturing. The risk of RAA rupture increases during pregnancy and delivery.

**Case presentation:**

A 29-year-old woman at 36 weeks and 5 days of gestation presented with severe back and abdominal pain. No fetal movements were identified. Cesarean section (C/S) was performed due to severe fetal bradycardia. No signs of placental abruption or abnormalities of the placenta were apparent intraoperatively, but gross hematoma was identified intraoperatively in the left retroperitoneal space. To evaluate persistent hypotension and retroperitoneal hematoma, contrast-enhanced computed tomography was performed and revealed ruptured RAA in the left kidney. Transcatheter arterial embolization (TAE) was performed.

**Conclusions:**

This case report describes fetal dysfunction caused by RAA rupture and controlled by TAE.

## Background

Renal artery aneurysms (RAAs) in pregnancy are uncommon, with most found after rupturing. It has been reported that the prevalence of visceral artery aneurysm (VAA) is 0.01–2% of the population [1, 2]. However, the number of undetected VAAs may be much larger, as VAAs are usually asymptomatic. Risk factors for rupture include hypertension, aneurysm size ≥ 2 cm, non-calcified aneurysm, and pregnancy [3].

Dissection or aneurysm rupture during pregnancy is considered more likely to occur. Although RAA occupy approximately 0.01% of VAA and there are only 32 cases reported over the past 20 years [4], over 50% of ruptured RAAs under 40 years old are reportedly related to pregnancy [5]. We reported a case of emergency cesarean section due to fetal bradycardia of unknown origin, which turned out to be caused by renal artery aneurysm rupture.

## Case presentation

A 29-year-old woman (height, 155 cm; weight, 56 kg) at 36 weeks and 5 days of gestation presented due to severe back and abdominal pain and the absence of fetal movement. The patient had a medical history of Sjögren syndrome and hypertension. On arrival, although ultrasound (US) did not reveal uterine rupture or placental abruption, fetal heart rate (HR) was 80 beats/min, indicating bradycardia. Fetal status was non-reassuring due to the fact that bradycardia had developed, so the decision was made to immediately perform emergency cesarean section (C/S), and we selected general anesthesia as the fastest method of induction. Surgery was started 16 min after arrival. Preoperative blood testing showed a hemoglobin (Hb) level of 10.3 g/dL. When the patient entered the operating room, blood pressure was 70/40 mmHg and heart rate was 90 beats/min. Thiopental (250 mg) was used for induction, and anesthesia was maintained using oxygen, nitrous oxide, and sevoflurane. After delivery, maintenance was performed using oxygen, air, propofol 200–300 mg/h, and remifentanil 0.2 μg/kg/min. The fetus was delivered 3 min after skin incision, followed by the placenta 2 min later. Placental abruption was considered unlikely, since no hematoma was observed in the placenta during the operation. A large hematoma was identified in the left retroperitoneum while ensuring hemostasis. Blood testing after delivery of the fetus revealed severe anemia (Hb, 4.7 g/dL). Although the mother was given 6 U of red blood cells and 4 U of fresh frozen plasma, Hb remained at 6.6 g/dL. So, after skin closure, while receiving intravenous (IV) fluids, transfusion, and vasopressors, the patient was sent for computed tomography (CT) angiography to search for the origin of bleeding under maintenance of general anesthesia.

**Fig. 1:**
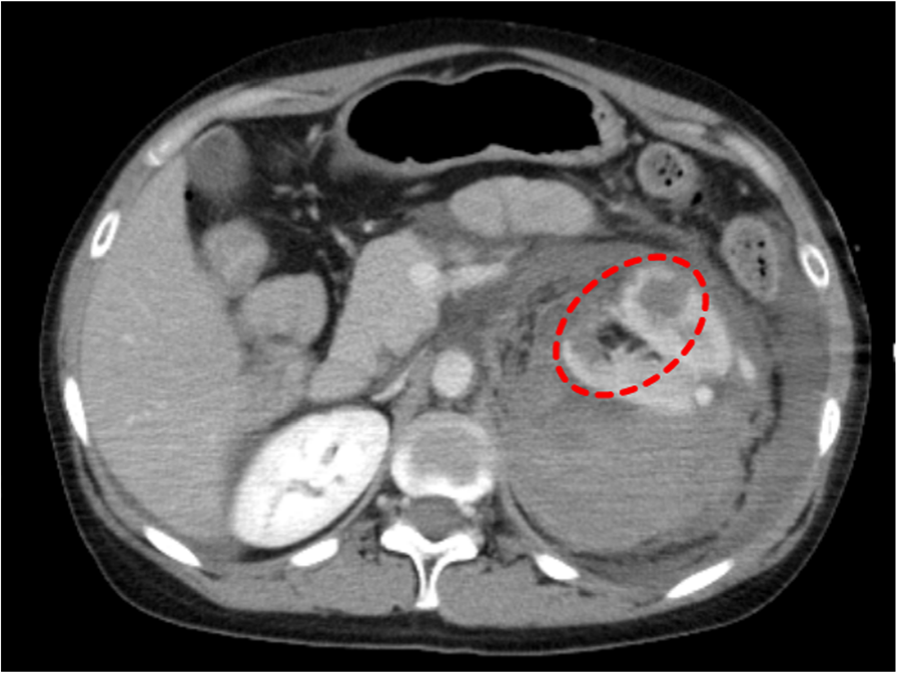
Contrast-enhanced CT. Contrast-enhanced CT shows two potential sites of intrarenal rupture of arteries (circle)

**Fig. 2:**
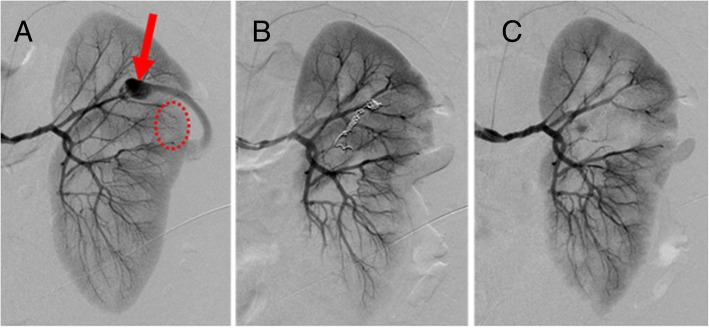
Transcatheter arterial embolization (TAE). **a** Angiography reveals two locations of contrast leakage in the left upper kidney before TAE. Arrow indicates renal artery aneurysm (RAA) and extravasation of contrast media. Circle indicates defect of contrast media in the kidney parenchyma. **b** Coil embolization is performed for RAA. **c** When contrast perfusion in the kidney parenchyma is lost, embolization is performed with gelatin sponge

Contrast-enhanced CT was performed to identify bleeding points, and contrast leakage was identified in the left kidney (Fig. 1). Renal artery angiography after CT imaging revealed contrast leakage from an intrarenal aneurysm in the left upper renal artery. Other points of leakage were unclear, but part of the kidney parenchyma showed a loss of contrast perfusion. Coil embolization was performed for the first leakage site, at the intrarenal bifurcation. Considering the results of the previous CT, embolization was performed with gelatin sponge for the second site (Fig. 2).

After admission to the intensive care unit (ICU), no signs of hemorrhage were seen and the patient remained stable. Her trachea was extubated 2 h later, and she was discharged from the ICU on postoperative day (POD) 1. The mother continued to have a fever of 38 °C for 2 weeks, and infection from retroperitoneal hematoma was suspected, although blood culture yielded negative results. Sulbactam and ampicillin were administered. Hospitalization was extended for another few days to ensure everyday life did not cause re-expansion of the hematoma. She was discharged from hospital on POD 22. Back pain persisted for 2 weeks postoperatively, but was relieved using a nonsteroidal anti-inflammatory drug. No renal dysfunction was seen after performing TAE. Blood pressure was well-controlled at the time of discharge, and the hematoma was shrinking on CT. Angiography to search for additional aneurysms was scheduled for 6 months after discharge.

The female neonate was born with a weight of 2414 g and a height of 45 cm. No spontaneous breathing was present at birth, HR was < 100 beats/min, and umbilical arterial blood gas analysis showed acidemia (pH, 6.887). Mask ventilation increased HR and improved skin color. After starting to cry and breathe spontaneously, the baby was sent to the neonatal ICU on free-flow O_2_ with fraction of inspiratory oxygen at 40%. Apgar score was 2 at 1 min after birth and 9 at both 5 and 10 min after birth. Oxygen was stopped the next day, and the newborn remained stable. She received phototherapy for neonatal jaundice at 3 days old. Then she moved to the growing care unit at 4 days old, and she was able to be discharged from the hospital.

## Discussion

In this case, cesarean section (C/S) was performed for fetal bradycardia of unknown origin. Retroperitoneal hematoma was found intraoperatively and turned out to be caused by the rupture of a RAA. This case was rare and unique, in that RAA rupture in a pregnant woman was the cause of prolonged hypotension and retroperitoneal hematoma. Fetal bradycardia likely resulted from blood loss and decreased perfusion to the placenta.

The prevalence of visceral artery aneurysm (VAA) has been reported as 0.01–2% of the population [1, 2]. However, the number of undetected VAAs may be much larger, as VAAs are usually asymptomatic. VAAs can be found in the following locations: splenic artery, 60%; hepatic artery, 20–50%; superior mesenteric artery, 5.5–6%; celiac artery, 4%; gastric and gastroepiploic arteries, 4%; jejunal, ileal, and colic arteries, 3%; pancreaticoduodenal and pancreatic arteries, 2%; gastroduodenal artery, 1.5%; inferior mesenteric artery, < 1%; and renal artery, 0.01–0.09% [2, 3, 6]. Risk factors for rupture include hypertension, aneurysm size ≥ 2 cm, non-calcified aneurysm, and pregnancy [3]. The mortality rates for maternal and fetal death due to VAA rupture are 70–75% and 90–95%, respectively. Since 24–45% of VAAs rupture during the last trimester and the early phase after delivery, these periods are particularly hazardous [2]. Although RAA comprises approximately 0.01% of VAAs and only 32 cases have been reported over the past 20 years [4], over 50% of ruptured RAAs in patients under 40 years old are reportedly related to pregnancy [5].

Rupture of RAA likely caused lumbar and abdominal pain, but pressure from the gravid uterus limited the severity of hemorrhage, and the mother did not experience severe shock right away. After delivery, decreased pressure from the uterus caused an increase in hemorrhage from the ruptured RAA, leading to enlargement of the retroperitoneal hematoma and persistent hypotension despite transfusion and administration of IV fluids and vasopressors.

At the time of starting surgery, maternal hypotension and tachycardia were indicated to have resulted from hemorrhage. Thorough examination by US, not just of the uterus and placenta, could have detected RAA or retroperitoneal hematoma on arrival. However, continuous fetal bradycardia did not allow any delay in emergency C/S, so the origin was detected intraoperatively. Although preoperative US yielded no significant results, delivering the baby may have made the hematoma more prominent as uterine pressure on the origin of hemorrhage was released.

Dissection or aneurysm rupture during pregnancy is considered more likely to occur. Hemodynamic stresses and hormonal changes associated with pregnancy could lead to changes in arterial structure and integrity. Various theories have been suggested in terms of these changes. Some previous studies have referred to possibilities including increased outflow resistance of arteries due to the increase in circulating blood and compression by the gravid uterus, vasodilation induced by estrogen or nitric oxide (NO) [7], histological changes in reticulin fibers or elastic fibers, and changes in smooth muscle cells [8, 9]. Underlying diseases related to the formation of VAA include vasculitis, fibromuscular dysplasia, syphilis, hypertension, atherosclerosis, and trauma [2, 10]. Sjögren syndrome, as seen in this case, is not typically associated with VAA.

In conclusion, we reported a case of emergency cesarean section due to fetal bradycardia of unknown origin, which turned out to be caused by renal artery aneurysm rupture. Non-reassuring fetal status with maternal hypotension may reveal underlying retroperitoneal hemorrhage. Since preoperative US did not show placental abruption or uterine rupture in this case, other causes of non-reassuring fetal status should have been considered. Pregnancy increases the risk of VAA rupture, and several reports have described VAA rupture during the perinatal period. Rupture of the VAA should be considered when sudden-onset abdominal pain and acute hemodynamic changes are seen during pregnancy. When emergency C/S is indicated, the focus tends to be on delivering the fetus immediately, rather than finding the cause. However, rupture of aneurysms should not be forgotten.

## Abbreviations

C/S: Cesarean section
CT: Computed tomography
Hb: Hemoglobin
HR: Heart rate
ICU: Intensive care unit
IV: Intravenous
NO: Nitric oxide
NSAID: Nonsteroidal anti-inflammatory drug
POD: Postoperative day
RAA: Renal artery aneurysm
TAE: Transcatheter arterial embolization
US: Ultrasound
VAA: Visceral artery aneurysm

## References

[1] Pitton MB, Dappa E, Jungmann F, Kloeckner R, Schotten S, Wirth GM (2015). Visceral artery aneurysms: incidence, management, and outcome analysis in a tertiary care center over one decade. Eur Radiol.

[2] Juntermanns B, Bernheim J, Karaindros K, Walensi M, Hoffmann JN (2018). Visceral artery aneurysms. Gefasschirurgie..

[3] Soliman KB, Shawky Y, Abbas MM, Ammary M, Shaaban A (2006). Ruptured renal artery aneurysm during pregnancy, a clinical dilemma. BMC Urol.

[4] Hellmund A, Meyer C, Fingerhut D, Müller SC, Merz WM, Gembruch U (2016). Rupture of renal artery aneurysm during late pregnancy: clinical features and diagnosis. Arch Gynecol Obstet.

[5] Barrett JM, Van Hooydonk JE, Boehm FH (1982). Pregnancy-related rupture of arterial aneurysms. Obstet Gynecol Surv.

[6] Messina LM, Shanley CJ (1997). Visceral artery aneurysms. Surg Clin North Am.

[7] Nolte JE, Rutherford RB, Nawaz S, Rosenberger A, Speers WC, Krupski WC (1995). Arterial dissections associated with pregnancy. J Vasc Surg.

[8] Konishi Y, Tatsuta N, Kumada K, Minami K, Matsuda K, Yamasato A (1980). Dissecting aneurysm during pregnancy and the puerperium. Jpn Circ J.

[9] Manalo-Estrella P, Barker AE (1967). Histopathologic findings in human aortic media associated with pregnancy. Arch Pathol.

[10] JCS Joint Working Group. Guideline for management of vasculitis syndrome. The Japan Circulation Society. 2017. http://www.j-circ.or.jp/guideline/pdf/JCS2017_isobe_h.pdf. Accessed 23 Mar 2018. 10.1253/circj.cj-88-0007 21263196

